# Influence of Two DNA Repair Pathway Polymorphisms in Colorectal Cancer Risk in Southwest Iran

**DOI:** 10.31557/APJCP.2020.21.7.1919

**Published:** 2020-07

**Authors:** Seyed Mohammad Hosseini, Javad Mohammadiasl, Abdolhasan Talaiezadeh, Rahim Alidadi, Mahdi Bijanzadeh

**Affiliations:** 1 *Department of Medical Genetics, Faculty of Medicine, Ahvaz Jundishapur University of Medical Sciences, Ahvaz, Iran. *; 2 *Cancer, Environmental and Petroleum Pollutants Research Center, Ahvaz Jundishapur University of Medical Sciences, Ahvaz, Iran. *; 3 *Department of General Surgery, School of Medicine, Ahvaz Jundishapur University of Medical Sciences, Ahvaz, Iran. *

**Keywords:** Polymorphism, colorectal cancer, restriction fragment length polymorphism

## Abstract

**Objective:**

X-ray cross-complementing group 1 (*XRCC1*) and 8 Oxo guanine DNA-glycosylase 1 (OGG1) genes are implicated in the repair of single-stranded breaks (SSBRs) and base excision repair (BER) pathways. Common polymorphisms in DNA repair genes are supposed to decrease the capability of DNA repair and cause genetic instability. This study was designed to investigate the association between *XRCC1* (*rs25487*) and *OGG1* (*rs1052133*) polymorphisms and susceptibility to colorectal cancer (CRC) in the Ahvaz city, south-west Iran.

**Methods:**

This case- control study comprised 150 patients and 150 controls that were selected from 2 educational hospitals in Ahvaz. They were matched for age and gender, and their genotyping was carried out by polymerase chain reaction-restriction fragment length polymorphism (PCR-RFLP).

**Results:**

Our results indicate that the frequency of the Gln (A) allele of *XRCC1* (*rs25487*) is significantly higher in colorectal cancer patients, compare to controls (p = 0.01, OR: 1.54, 95% CI 1.9–13.3). Significant increased risk of cancer was observed in *XRCC1* (*rs25487*) genotypes (p = 0.001 OR: 5.3, 95% CI 1.9–14.2 for Gln / Gln), while no association was found between *OGG1 *(*rs1052133*) and colorectal cancer risk (p = 0.6).

**Conclusion:**

Our study suggests that *XRCC1* (*rs25487*) polymorphism might be associated with an increasing risk of CRC in Ahvaz. It also demonstrates positive correlation between the *XRCC1* (*rs25487*) genotypes and demographic characteristics, such as smoking and increased age in patients and control groups.

## Introduction

Colorectal cancer (CRC) is one of the most common malignancies and the fourth leading cause of death by cancer in the world (Arnold et al., 2017). The incidence of colorectal cancer seems to have increased in Iran: occurrence of CRC has increased with age and highest rates have been observed in the older peoples (Safaee et al., 2012). CRC is a complex disorder caused by the composite of genetic susceptibilities and environmental factors. Diet, smoking, and alcohol consumption are environmental factors that have shown association with CRC risk (Pardini et al., 2008). Candidate genes considered susceptible to CRC are implicated in tumor suppressor genes, oncogenes, methylation mechanism, and genes involved in DNA repair pathways (de Jong et al., 2002). In mammalian cells, four important mechanisms of DNA repair have been recognized: nucleotide excision repair (NER), base excision repair (BER), double-strand break repair, and mismatch repair (Karam et al., 2016). Some common genetic polymorphisms in DNA repair genes are supposed to decrease capability of DNA repair and genetic instability that may contribute to the development of cancers such as CRC (Wood et al., 2001). Among the established polymorphisms of the DNA repair genes, the X-ray cross-complementing group 1 (*XRCC1*) and 8 Oxo guanine DNA-glycosylase 1 (*OGG1*) polymorphisms have been frequently studied as potentially related with susceptibility to the incidence of several cancers (Simonelli et al., 2012; Nissar et al., 2014). The DNA repair gene *XRCC1* is implicated in the repair of single-stranded breaks (SSBRs) and base excision repair (BER) that has been mapped to the human chromosome region 19q13 (Mohrenweiser et al., 1989; Huang et al., 2015). Another DNA repair gene that has an essential role in base excision repair is* OGG1 *that is located in the chromosome region 3p26. This glycosylase enzyme removes damaged bases by way of reactive oxygen. *XRCC1* has unknown enzymatic activity and is considered to operate as a scaffold protein for base excision repair activities (Hung et al., 2005). *XRCC1* has been reported to interact with other crucial proteins such as DNA polymerase β, DNA ligase III, poly (ADP-ribose) polymerase, APE1, and OGG1 in base excision repair pathway (Fan et al., 2004; Nazarkina et al., 2007). One of the common genetic polymorphism in the *XRCC1* gene is Arg399Gln (rs25487) at amino acid residue 399 (Arg to Gln) of exon 10: this substitution can alter the functionality of XRCC1 protein and damage its DNA repair activity (Monaco et al., 2007). The *OGG1* is an important DNA repair gene that encodes a DNA glycosylase for removal of the 8-oxoG adduct of oxidatively damaged DNA (Weiss et al., 2005). Although many polymorphisms have been reported, Ser326Cys (rs1052133) polymorphism at the codon 326 of exon 7 (Ser to Cys) in *OGG1* gene is the most studied polymorphism (Boiteux and Radicella, 2016). It has been reported to affect the OGG1 function and is linked to increased risk of different cancers (Kohno et al., 1998, Wang et al., 2015). Therefore, this study was designed to investigate the association between the polymorphisms of DNA repair genes, including *XRCC1* (*rs25487*) and *OGG1* (*rs1052133*) given their susceptibility to colorectal cancer in the Ahvaz city, south-west Iran.

## Materials and Methods

*Study population*

This study comprised 150 patients (82 males and 68 females) with mean age 54.89 ± 13.5 SD and 150 controls (70 males and 80 females) with mean age 55.09 ± 11.5 SD. Informed consent was obtained from all participants for their participation in the study. The ethics committee of the Jundishapur University of Medical Sciences approved the study. An organized questionnaire was applied to collect information from study subjects about lifestyle habits (smoking, diet, and alcoholism). Patients include subjects with positive colonoscopic findings for malignancy and histologically accepted as carcinomas of colorectal. The control subjects were picked among individuals who refers to hospital with no evidence and history of cancer. Patients and controls were matched were age and gender. Our exclusion criteria for control group are personal history of all cancers and chronic diseases such as hypertension, diabetes mellitus, and nephritic disorder. Patients who were receiving neoadjuvant chemotherapy and suffered from autoimmune and metabolic diseases were excluded from the study. 

*DNA extraction and genotyping*

DNA for genotyping was isolated from whole blood samples (5 ml collected into EDTA tubes) taken from CRC patients and control subjects. DNA extraction was performed by DNA Extraction Kit (Favorgen Biotech, Taiwan) conforming to the manufacturer’s procedure.

Genotyping was carried out by restriction fragment length polymorphism (RFLP) after polymerase chain reaction (PCR); detailed information of procedures for PCR-RFLP is shown in Table 1.

*Statistical analysis*

The differences of genotype frequencies of the *XRCC1* and *OGG1* gene polymorphisms between clinical features within the cases and controls were analysed using the Chi-square test. Association between these polymorphisms and CRC was calculated with 95% odds ratios (OR) and confidence intervals (CI). p value < 0.05 was considered statistically significant. Adjusted Odds ratio (OR), 95% confidence intervals (CIs), and p-values were determined by logistic regression in order to evaluate the association between each genotype and risk of CRC. The binary logistic regression model included age, gender, and smoking. All statistical analysis was performed using the SPSS software for Windows version 18.0 (SPSS, Chicago, IL, USA).

## Results

**Figure 1:**
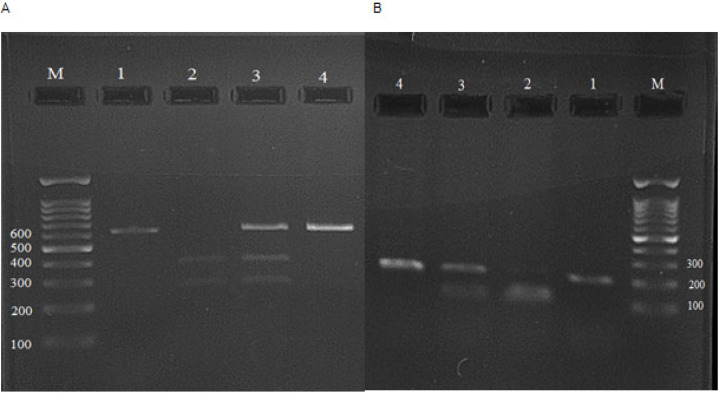
Representative Electrophoresis Gels Obtained by PCR-RFLP for the *XRCC1 (rs25487)* and *OGG1 (rs1052133)* polymorphisms. M is DNA marker ladder contains a 100 base pair (bp) marker and lane 1 is undigested PCR-amplified product. The left panel is for the *XRCC1 (rs25487) *polymorphism: lane 2 is genotype AA for Gln / Gln: 375 bp, 240 bp; lane 3 is genotype AG for Arg/Gln: 615 bp, 375 bp, 240 bp and lane 4 is genotype GG for Arg/Arg: 615 bp. The right panel is for the *OGG1 (rs1052133)* polymorphism: lane 2 is genotype GG for Cys/Cys: 100 bp; lane 3 is genotype CG for Ser/Cys: 100 bp, 200 bp; and lane 4 is genotype CC for Ser/Ser: 200 bp

Out of 150 patients, 49 patients (32.7%) had less than 50 years and 101 (67.3%) had more than 50 years old, compare to 46 controls (30.7%) less than 50 years and 104 controls (69.3%) more than 50 years (p= 0.7). 82 patients were male and 68 were female (54.7% and 45.3% respectively), while 70 healthy controls were male (46.7%) and 80 were female (53.3%). 40 out of 150 patients were smoker (26.7%) and 44 out of 150 controls (29.3%) were smoker. There were no significant differences in parameters of mean age (p= 0.8), gender (p= 0.16) and smoking (p= 0.7) between the patients and controls. In 85 patients cancers located in colon (56.7%) and 65 patients suffered from rectal cancer (43.3%); 74 cancers were in stage I and II (49.3%) and 76 in stages III and IV (50.7%). Among 18 patients metastasis were detected (12%), while in 132 ones, metastasis did not reported (88%). Representative PCR-based restriction analyses for the *XRCC1* (*rs25487*) and* OGG1* (*rs1052133*) polymorphisms are shown in Figure 1.

The distributions of the genotype and allele frequencies for *XRCC1* (*rs25487*) and *OGG1 *(*rs1052133*) polymorphisms in patient and control groups are represented in Table 2. We found that the frequency of the Gln (A) allele of *XRCC1* (*rs25487*) was significantly higher in CRC patients than in controls (55.0% vs. 65.3%, p = 0.01, OR: 1.54, 95% CI 1.1–2.1). Also, frequency of the Gln/Gln genotype of *XRCC1* (*rs25487*) was higher in CRC patients than in controls. No significant association was found between the Cys allele and the Ser/Cys genotype of *OGG1* (*rs1052133*) in CRC patients as compared with controls. Furthermore, the associations of genetic models with CRC risk in each polymorphism were evaluated. For *XRCC1* (*rs25487*) polymorphism, significantly increased cancer risk was seen in the recessive genetic model. Influence of interaction between demographic and clinical characteristics of *CRC* and *XRCC1* (*rs25487*) (Table 3) and *OGG1 *(*rs1052133*) (data not shown) polymorphisms have been displayed. Our results demonstrate a positive correlation between *XRCC1* (*rs25487*) genotypes and some demographic characteristics in patient and control groups, such as smoking and age more than 50 years. Simultaneously, no differences in frequencies of the *OGG1* (*rs1052133*) genotypes were found in patient and control groups in all demographic features. In addition, association between *XRCC1* gene polymorphism and pathologic characteristics is shown in Table 4, though for *OGG1* (*rs1052133*) polymorphism the data is not shown.

**Table 1:** Details of PCR and RFLP Procedures and Observed Products

Genotype	Primers (forward and reverse)	PCR conditions	PCR product	Restriction enzyme	Restriction products
OGG1 (*rs1052133) *	5-GGAAGGTGCTTGGGAAT-3 5-ACTGTCACTAGTCTCACCAG-3	25 µl of PCR mixture:8ul master mix with 3 µl DNA and 14 µl dw. 35 cycles: 95 ^o^C 1 min, 60 ^o^C 1 min, 75 ^o^C 1 min	200 bp	*Fnu4HI*	Ser/Ser: 200 bp Ser/Cys: 100 bp, 200 bp Cys/Cys: 100 bp +100 bp
XRCC1 *(rs25487)*	5-TTGTGCTTTCTCTGTGTCCA-3 5-TCCTCCAGCCTTTTCTGATA-3	25 µl of PCR mixture: 8 µl master mix with 3 ul DNA and 14 µl dw. 35 cycles: 95 ^o^C 1 min, 60 ^o^C 1 min, 75 ^o^C 1 min	615bp	*Msp1*	Gln / Gln: 375 bp, 240 bp Arg/Gln: 615 bp, 375 bp, 240 bp Arg/Arg: 615 bp

^1^Distilled water

**Table 2:** Comparison of Genotypes and Alleles Frequencies of each Polymorphism between Patients and Controls Groups

Genotype/Allele	Patients	Controls	OR (95% CI)^2^	*P*-value	Adjusted^3^	
	N=150 (%)	N=150 (%)			OR (95% CI)	*P*-value
*XRCC1 *Arg399Gln (*rs25487*)						
Arg/Arg (GG)	39 (26.0%)	52 (34.7%)	1 (reference)			
Arg/Gln (GA)	87 (58.0%)	92 (61.3%)	1.2 (0.7-2.09)	0.3	1.33 (0.76-2.35)	0.3
Gln / Gln (AA)	24 (16.0%)	6 (4.0%)	**5.3 (1.9-14.2)**	**0.001**	**7.20 (2.3-22.1) **	**0.001**
Arg (G)	135 (45.0%)	104 (34.7%)	1 (reference)			
Gln (A)	165 (55.0%)	196 (65.3%)	**1.54 (1.1-2.1)**	**0.01**		
AA vs GG+GA	127 (84.7%)	144 (96.0%)	**4.34 (1.71-11.02)**	**0.001**	**5.3 (1. 9-13.3) **	**0.001**
*OGG1* Ser326Cys (*rs1052133*)						
Ser/Ser (CC)	91 (48.9%)	95 (51.1%)	1 (reference)			
Ser/Cys (CG)	57 (51.4%)	54 (48.6%)	1.1 (0.6-1.7)	0.6	1.03 (0.7-1.7)	0.2
Cys/Cys (GG)	2 (2.2%)	1 (1.0%)	2.08 (0.2-23)	0.5	3.08 (0.3-36)	0.3
Ser (C)	239 (79.7%)	244 (81.3%)	1 (reference)			
Cys (G)	61 (20.3%)	56 (18.7%)	1.09 (0.7-1.6)	0.6		

^2^Crude OR; ^3^Adjusted for gender, age, smoking; Significant values are shown in bold.

**Table 3:** Influence of Interaction between Clinical and Demographics Characteristics of CRC and *XRCC1 (rs25487)* Gene Polymorphism

Parameters	Patients (number)	controls (number)	OR (CI95%)^4^	*P*-value	OR(CI95%)^5^	*P*-value
Gender						
Man	82	70				
Arg/Arg	28	26	1 (reference)		1 (reference)	
Arg/Gln	38	41	1.2 (0.5-2.3)	0.6	0.9 (0.4-2)	0.9
Gln / Gln	16	3	4.9 (1.3-18)	0.02	8.6 (1.7-41)	0.007
AA vs GG+GA	66	67	2.3 (1.2-4.4)	0.01	8.1 (2-32)	0.003
Woman	68	80				
Arg/Arg	11	26	1 (reference)		1 (reference)	
Arg/Gln	49	51	0.4 (0.1-0.9)	0.04	2.1 (0.9-4.7)	0.07
Gln / Gln	8	3	6.3 (1.4-28.3)	0.01	6.8 (1.2-36)	0.02
AA vs GG+GA	60	7	1.8 (0.9-3.5)	0.07	3.8 (0.8-17)	0.07
Smoking						
Yes	40	44				
Arg/Arg	9	17	1 (reference)		1 (reference)	
Arg/Gln	16	25	1.2 (0.4-3.3)	0.7	1.1 (.03-4)	0.7
Gln / Gln	15	2	**14.1 (2.6-76)**	**0.002**	**21 (3-140)**	**0.002**
AA vs GG+GA	25	42	**12.6 (2.6-59)**	**0.001**	**13 (3-65)**	**0.002**
No	110	106	1 (reference)		1 (reference)	
Arg/Arg	30	35	1.2 (0.6-2.2)	0.4	1.5 (0.8-2.9)	0.1
Arg/Gln	71	67	2.6 (0.7-9.3)	0.1	3.5 (0.8-14.8)	0.08
Gln / Gln	9	4	2.2 (0.6-7.6)	0.1	2.1 (0.6-7.2)	0.2
AA vs GG+GA	80	71	2.2 (0.6-7.6)	0.1	2.1 (0.6-7.4)	0.2
age						
<50	49	46				
Arg/Arg	15	17	1 (reference)		1 (reference)	
Arg/Gln	27	29	1.05 (0.4-2.5)	0.9	1.1 (0.3-2.6)	0.9
Gln / Gln	7	0	-----------		-----------	
AA vs GG+GA	42	46	-----------		-----------	
>50	101	104				
Arg/Arg	24	35	1 (reference)		1 (reference)	
Arg/Gln	60	63	1.3 (0.7-2.6)	0.3	1.5 (0.7-3)	0.2
Gln / Gln	17	6	**4.1 (1.4-11.1)**	**0.009**	**6.3 (1.8-20.6)**	**0.003**
AA vs GG+GA	84	98	**3.3 (1.2-8.7)**	**0.01**	**3.4 (1.2-9.6)**	**0.01**

^4^ Crude OR; ^5^ Adjusted for gender, age, smoking; Significant values are shown in bold.

**Table 4:** Association between *XRCC1* Gene Polymorphism and Tomur Characteristics

Variables	Cases (n=150)		
	Group I	Group II	OR (95% CI); p
Tumor location	Colon = 49 (42.9%)	Rectum = 65 (57.1%)	
Arg/Arg; n = 39	21 (53.8%)	18 (46.2%)	1 (Ref)
Arg/Gln; n = 87	49 (56.3%)	38 (43.7%)	0.9 (0.4–1.9); 0.5
Gln / Gln; n = 24	15 (62.5%)	9 (37.5%)	0.7(0.2–1.9); 0.7
AA vs GG+GA; n = 126	70 (55.6%)	56 (44.4%)	0.7 (0.3–1.8); 0.5
Metastasis	Yes = 18 (12%)	No = 132 (88%)	
Arg/Arg; n = 39	4 (10.3%)	35 (89.7%)	1 (Ref)
Arg/Gln; n = 87	8 (9.2%)	79 (90.8%)	1.1 (0.3–3.9); 0.8
Gln / Gln; n = 24	6 (25%)	18 (75%)	0.3 (0.08–1.3); 0.1
AA vs GG+GA; n = 126	12 (9.5%)	114 (90.5%)	**0.3 (0.1–0.9); 0.04**
Stage	I+II = 74 (49.3%)	III+IV = 76 (50.7%)	
Arg/Arg; n = 39	25 (64.1%)	14 (35.9%)	1 (Ref)
Arg/Gln; n = 87	39 (44.8%)	48 (55.2%)	**2.1 (1–4.7); 0.04**
Gln / Gln; n = 24	10 (41.7%)	14 (58.3%)	2.5 (0.8–7.09); 0.08
AA vs GG+GA; n = 126	64 (50.8%)	62 (49.2%)	1.4 (0.5–3.4); 0.4

Significant values are shown in bold

## Discussion

The DNA repair system has an important role in conserving genetic stability. Polymorphisms in DNA repair genes can decrease the repair capacity of DNA, which may lead to development of different cancers including CRC (Goode et al., 2002). DNA damage can be caused by endogenous oxidation, deamination, or alkylation, these are repaired mainly by the base excision mechanism. The *XRCC1* and *OGG1* genes encode proteins that play critical roles in the base excision repair pathway (Karahalil et al., 2012). In this study, we aim to test the hypothesis that whether polymorphism in these genes influences the risk of CRC. The outcomes of some investigation about the association of *XRCC1* (*rs25487*) polymorphism with colorectal cancer risk have been contradictory. For example, two recent studies indicated association with increased risk of colorectal cancer (Karam et al., 2016; Abu Halim et al., 2016), while a case-control study in Sweden found no association with CRC risk (Salimzadeh et al., 2020). Also, other findings about the association of *OGG1* (*rs1052133*) polymorphism with susceptibility to CRC remain controversial (Lai et al., 2016; Zou et al., 2016). The results of our study demonstrate that the frequency of the Gln allele is significantly higher in CRC patients than in controls and is associated with the risk of colorectal cancer. Individuals with 399Gln homozygote genotype have more than 5 folds greater risk of colorectal cancer than those with the homozygous 399Arg genotype. Significant interactions were found between this polymorphism and smoking in smoker patients as compared to smoker controls and also with age more than 50 years. Our findings are consistent with the results of Fouad et al. in Egypt: they observed increased risk of colorectal cancer associated with the 399Gln allele (Fouad et al., 2017). In contrast, a study in the Italian population reported no differences among the *XRCC1* (*rs25487*) allele and the risk of colorectal cancer (Improta et al., 2008). 

The biological interaction between *XRCC1* polymorphism and smoking in colorectal cancer risk is probable. Cigarette smoking is a plentiful source of genotoxins such as reactive oxygen species and chemical carcinogens that can cause single and double-stranded breaks and base adduct formation (Stern et al., 2007). Smoking has been shown to be associated with increased risk of colorectal cancer consistent with our study (Weiss et al., 2005; Tsong et al., 2007).

Our data analysis revealed no significant difference between CRC patients and controls in the genotypic and allelic frequencies of the *OGG1* (*rs1052133*) polymorphism. Zou et al., (2016) in a meta-analysis found no major difference in that genotype distribution between patients and controls, this confirms our results. In contrast to our findings, *OGG1* (*rs1052133*) polymorphism had significant association with increased risk of CRC in the Polish population according to Kabzinski et al., (2018). Based on our results, *XRCC1* polymorphism may be considered a substantial biomarker for cancer screening in high risk persons and for supporting these patients by prophylactic interventions and specific therapeutic methods. Due to our limited sample size, further studies with large sample size across diverse ethnic populations should be performed to elucidate the association between *XRCC1* (*rs25487*) and *OGG1* (*rs1052133*) polymorphisms and CRC in the Iranian population.
